# Peak flow rate and death due to coronary heart disease: 30-year results from the Northwick Park Heart cohort study

**DOI:** 10.1136/openhrt-2014-000164

**Published:** 2014-10-08

**Authors:** Tim C Clayton, Tom W Meade, Elizabeth L Turner, Bianca L De Stavola

**Affiliations:** 1 Department of Medical Statistics, London School of Hygiene and Tropical Medicine, London, UK; 2 Department of Non-Communicable Disease Epidemiology, London School of Hygiene and Tropical Medicine, London, UK; 3 Department of Biostatistics and Bioinformatics, Duke University School of Medicine, Durham, USA; 4 Duke Global Health Institute, Duke University, Durham, USA

## Abstract

**Objective:**

Numerous studies have reported that chronic obstructive pulmonary disease or impaired lung function are associated with later coronary heart disease (CHD). However, it is unclear if lung function is an independent risk factor, as many of these studies have included only limited measures of other factors associated with CHD.

**Methods:**

In total 2167 men of all ages in the first Northwick Park Heart Study were followed for a median of 30 years. Cox regression models were used to assess the relationship between peak flow rate (PFR) and CHD mortality adjusted for potential confounders measured at baseline. Analyses allowed for missing data, and secondary analyses for repeat measures on some men and competing risks of CHD death.

**Results:**

There were 254 CHD deaths with some evidence of an association between PFR and CHD mortality. The adjusted HRs (95% CIs) from the lowest to the highest of four PFR quartiles were 1.53 (1.04 to 2.25), <430 L/min; 1.43 (0.99 to 2.08), 430 – <490 L/min; and 1.31 (0.93 to 1.86), 490 – <550 L/min; compared with the reference group of ≥550 L/min (trend test p=0.025). Other associations with CHD mortality were observed for systolic blood pressure (p<0.0001), body mass index (p=0.0002), smoking status (p=0.015), blood cholesterol (p=0.005), plasma fibrinogen (p=0.001) and high-risk ECG (p=0.021). There were no strong associations for factors V and VIII or platelet count.

**Conclusions:**

After allowing for a range of other risk factors associated with CHD, there was only limited evidence of a relation between PFR and CHD mortality.

**KEY MESSAGES**

**What is already known about this subject?**

- Impaired lung function is thought to be a risk factor for coronary heart disease.

**What does this study add?**

- While there is evidence of an association between respiratory function and coronary heart disease it is unlikely to be a strong risk factor.

**How might this impact on clinical practice?**

- It is important to deal with impaired respiratory function but it is unlikely to impact strongly on the risk of coronary heart disease to the extent suggested by other studies which indicated a doubling in the risk whereas our study, after adjusting for a range of risk factors, indicates an increased risk of approximately 40%.

## Introduction

There have been many reports that chronic obstructive pulmonary disease (COPD) or measures found in impaired pulmonary function such as peak flow rate (PFR) or forced expiratory volume (FEV) are associated with coronary heart disease (CHD) events.^1–16^ Consequently, impaired respiratory function is generally considered as a risk factor for CHD. Results similar to those for CHD have been reported for stroke.^17^ ^18^ Associations between respiratory function with the development of carotid atherosclerotic plaques^19^ and with central arterial stiffness have also been described. This report uses PFR as an index of respiratory function to assess the relationship with CHD mortality. The study's first advantage is that the results presented include data on some variables in the coagulation system influencing the thrombotic component of CHD that have not previously been reported in relation to PFR and COPD. Second, analyses of results have included methods to allow for imputation of missing values and also consideration of competing risks of non-CHD deaths. Third, repeat measurements have enabled assessments of the effects of changes over time using time-updated modelling techniques. Finally, participants have been followed up for a median duration of 30 years, so that the value or otherwise of data on PFR and CHD mortality over a longer time period than in other studies has been possible.

## Methods

This report is based on data from the first Northwick Park Heart Study (NPHS-1). Full details have been reported elsewhere.^20^

### Participants

Between 1972 and 1978, NPHS-I recruited men of all ages working at three industrial or occupational sites in North West London. The response to invitations to participate was about 80%. Methods for the clinical examination, laboratory measurements and clotting factor assays and follow-up have been described before.^20^ Follow-up for non-fatal events ended in 1986. However, follow-up for fatal events, with which this report is concerned, continued until June 2006, with notifications of deaths and their causes having been provided throughout the study by the Office for National Statistics. Blind assessments of causes of death have been made as before,^20^ based on information from general practitioners, hospitals and coroners.

As in previous reports of men in the NPHS-1 study, analyses are confined to white participants who had not previously had myocardial infarcts (but including 78 men with a history of definite or possible angina).^20^ Most men (1703/2167; 78%) had repeat blood samples taken at follow-up examinations between 1978 and 1984, at a median of 6.5 years after entry to the study. Comparison of baseline values for those who were or were not available for follow-up (excluding the 81 men who died within 6.5 years of entry to the study in order to include only those potentially available for follow-up) showed the former to be on average 4 years older, to use less alcohol and to have higher mean body mass index (BMI), blood cholesterol, red blood cell count, haemoglobin and packed cell volumes than those not followed up. Those not followed had lower age-adjusted all-cause mortality and similar CHD mortality compared with those followed up. Bearing these differences in mind, it has been possible to establish prospective data on the impact of PFR on cardiac mortality over time, using time-updated models to take account of the repeat measurements made.

### Measurements

With participants standing up, PFR was measured three times using the original Wright peak flow metre. The clinic nurse gave instructions on what was required, with a demonstration of the appropriate respiratory effort. With a brief rest interval between each of the three measures, the highest of these was recorded. Besides PFR itself and age and gender, characteristics recorded^20^ included smoking status, alcohol use, height and weight and hence BMI, blood pressure (average of three readings), social class, total cholesterol and triglycerides (fasting), and automated Coulter full blood count (details shown or noted in tables). Biological activities of clotting factors V, VII and VIII were assayed,^21–23^ and fibrinogen was measured by a clot weight method.^24^ Of the clotting factors, only results for factor VII and fibrinogen are shown, as those for V and VIII were not associated with CHD, although they were included in the statistical models to allow as fully as possible for any confounding effect on the relationship between PFR and CHD. ECG findings were coded according to the Minnesota code,^25^ and classified as indicating higher or lower risk of CHD according to the groupings established, and successfully validated, for the Medical Research Council (MRC) Hypertension Trial.^26^

NPHS-1 started in 1972, which was before the development of ethics committees in the UK. It was therefore not submitted to ethics committees. Those approached about the study were given a full verbal explanation of the reasons for it, its nature and what it would involve, and agreed to take part.

### Statistical analyses

PFR was considered in four categories: (1) <430 L/min (2) 430–<490 L/min (3) 490–<550 L/min and (4) ≥550 L/min. These categories were defined to include approximately a quarter of the CHD deaths in each. PFR was categorised in this way as there was no observed association between PFR and CHD mortality at higher levels of PFR. The characteristics of the patients at baseline and also for those who were followed up have been tabulated, and CHD death rates calculated. The primary analysis has focused on the association of PFR measured at baseline with CHD death, using Cox regression models. Unadjusted HRs for PFR were estimated in order to compare with adjusted HRs, and Kaplan-Meier curves showing the cumulative percentage of CHD death by PFR groups have been presented. All other analyses have been adjusted for age by setting the timescale in the Cox models to be current age, while also including age at baseline to allow that baseline characteristics may have been measured several years previously.^27^ A Cox regression model was developed for the primary analysis to allow for the confounding effect of other potential risk factors collected at baseline. Covariates of interest were included irrespective of the strength of evidence for their association with CHD mortality to more fully allow for any potential confounding due to these covariates. Data were missing for some variables, particularly for some of the laboratory measurements, although the numbers were low for baseline data (<5% for any one variable), being higher for follow-up data (<12%). In order to adjust for these missing data, multiple imputation techniques with chained equations were used, assuming missingness is at random, with 100 imputations using all covariates in the model including the outcome variable of CHD mortality and time in the study.^28^ Analyses were also conducted using a complete-case analysis (ie, those with values for all variables) and by imputing the median value for missing covariates to assess whether results were consistent with the findings from the primary analysis.

In order to assess whether the impact of PFR changed according to time in the study or with age, multivariable Cox models were fitted with interaction terms between PFR and (1) time in follow-up (≤15 or >15 years) or (2) current age (≤75 or >75 years) after first splitting the data according to the relevant timescales.

A secondary analysis was also conducted using information for those in whom a follow-up visit was conducted (acknowledging, as already indicated, that those followed up may not have been truly representative of the initial population studied). Time-updated Cox models accounting for repeat measurements of variables for those in whom a follow-up visit was conducted were used to consider age-adjusted and more fully adjusted HRs.

Finally, an analysis was conducted to consider whether there was any impact of competing risks due to deaths from non-cardiac causes using the method of Fine and Gray^29^ for competing risks regression.

All analyses were conducted using Stata V.13.0 (StataCorp. 2013. *Stata Statistical Software: Release 13*. College Station, Texas, USA: StataCorp LP).

## Results

**Figure 1:**
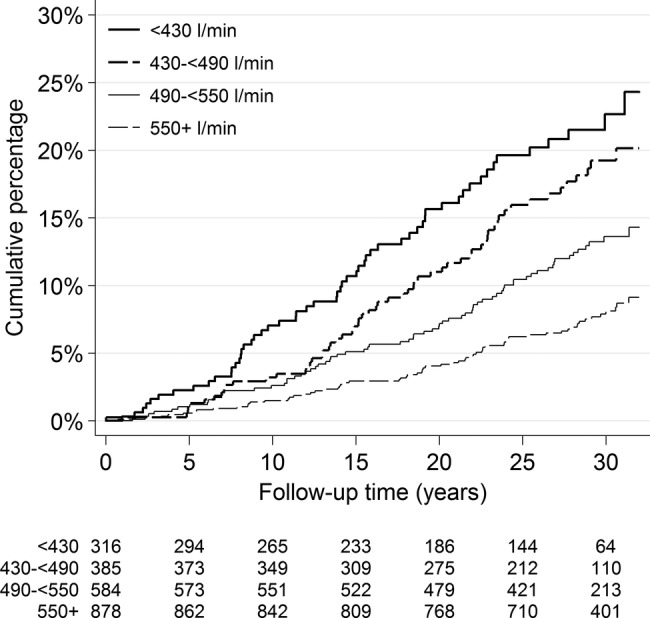
Kaplan-Meier curve of cumulative percentage of cardiac mortality by peak flow rate category (adjusted HRs (95% CI) compared with ≥550 L/min: (<430 L/min) 1.53 (1.04 to 2.25); (430 – <490 L/min) 1.43 (0.99 to 2.08); (490 – <550 L/min) 1.31 (0.93 to 1.86)).

In total, data for 2167 men were included in the analyses, and the men were followed for a median of 30 years. Table 1 shows that there were 254 cardiac deaths (rate 4.58/1000 person-years). In addition, 720 men died of non-cardiac causes over the follow-up period. Table 1 also shows that, as expected, advancing age is strongly associated with increasing HRs for CHD death. The unadjusted HRs increase with decreasing PFR (figure 1), although after adjustment for age the HRs move towards 1 (table 2). Table 2 gives results from the multivariable model and shows that after adjusting for age and other risk factors measured at baseline, including BMI and smoking, the impact of PFR on CHD is reduced further. The hazard for the lowest PFR quartile is 54% more than for the highest quartile, although the evidence for an association, though suggestive, is not strong (trend test p=0.025). There was an increased risk of CHD mortality with smoking, BMI, systolic blood pressure, cholesterol, fibrinogen and an abnormal ECG considered high risk by the Minnesota coding. NPHS-1 has not previously included results based on ECG data, and our results now show that ‘high-risk’ abnormalities are associated with increased CHD mortality, HR 1.58 (95% CI 1.11 to 2.27). The association of factor VII with CHD mortality is of marginal statistical significance (p=0.058). Other factors included in this analysis but with little evidence for an association with cardiac death were triglycerides, haemoglobin, red blood cell count, white blood cell count or packed cell volume (data not shown though referred to in table 2). Neither alcohol use nor social class appeared to be independent risk factors. There was no evidence that the association between PFR and CHD mortality changed over time of follow-up (interaction p value 0.79) or with age (interaction p=0.24).

**Table 1:** Characteristics and cardiac mortality of individuals at baseline and follow-up

Characteristic	Baseline			Follow-up		
		Cardiac death	Non cardiac death	Total	Cardiac death	Non cardiac death
	Total	N (rate)*	N*	N	N (rate)*	N*
Total	2167	254 (4.58)	1913	1703	192 (5.65)	1511
Peak flow rate (L/min)						
<430	316	57 (8.45)	259	346	50 (8.21)	296
430–<490	385	60 (6.57)	325	311	43 (7.12)	268
490–<550	584	71 (4.62)	513	401	41 (4.99)	360
≥550	878	66 (2.73)	812	559	42 (3.42)	517
Missing	4			86		
Mean (SD)		493 (90)	525 (91)		473 (109)	506 (102)
Age (years)						
<45	864	26 (1.01)	838	392	3 (0.31)	389
45–49	352	30 (3.13)	322	188	12 (2.85)	176
50–54	346	60 (7.22)	286	295	25 (3.94)	270
55–59	353	73 (9.61)	280	283	44 (8.09)	239
≥60	252	65 (15.53)	187	545	108 (12.94)	437
Missing	0			0		
Mean (SD)		54.7 (7.1)	45.2 (12.2)		60.6 (7.1)	52.4 (11.5)
ECG						
Low risk	1966	215 (4.22)	1751	1410	132 (4.54)	1278
High risk	201	39 (8.46)	162	293	60 (12.19)	233
Missing	0			0		
Previous angina						
No	2093	232 (4.30)	1861	1600	165 (5.11)	1435
Yes	74	22 (14.54)	52	103	27 (15.61)	76
Missing	0			0		
Smoking status						
Never	575	38 (2.37)	537	427	30 (3.23)	397
Ex-smoker	544	72 (5.24)	472	604	72 (6.13)	532
Current smoker	1048	144 (5.59)	904	672	90 (6.95)	582
Missing	0			0		
Body mass index (kg/m^2^)						
<22.5	409	20 (1.85)	389	237	17 (3.47)	220
22.5–<25	702	63 (3.39)	639	497	47 (4.65)	450
25–<27.5	648	88 (5.43)	560	516	53 (5.05)	463
27.5–<30	282	55 (7.94)	227	259	36 (7.03)	223
≥30	124	27 (9.12)	97	116	23 (10.42)	93
Missing	2			78		
Mean (SD)		26.3 (3.0)	24.9 (3.0)		26.4 (3.2)	25.4 (3.1)
Systolic blood pressure (mm Hg)						
<140	1154	82 (2.60)	1072	768	38 (2.27)	730
140–<160	652	79 (4.87)	573	532	67 (6.32)	465
160–<180	247	55 (9.89)	192	255	47 (10.52)	208
≥180	111	38 (18.09)	73	137	40 (20.03)	97
Missing	3			11		
Mean (SD)		153 (26)	137 (21)		160 (25)	143 (22)
Cholesterol (mmol/L)						
<5	403	22 (1.97)	381	284	16 (2.72)	268
5–<6	695	62 (3.42)	633	545	49 (4.39)	496
6–<7	584	78 (5.40)	506	431	63 (7.27)	368
≥7	365	79 (9.02)	286	242	33 (6.92)	209
Missing	120			201		
Mean (SD)		6.48 (1.23)	5.85 (1.15)		6.25 (1.06)	5.86 (1.11)
Factor VII (% of standard)						
<90	540	38 (2.58)	502	270	20 (3.51)	250
90–<110	675	69 (3.94)	606	491	46 (4.66)	445
110–<130	479	69 (5.88)	410	458	57 (6.15)	401
≥130	379	69 (7.62)	310	290	38 (6.57)	252
Missing	94			194		
Mean (SD)		117 (30)	106 (26)		115 (22)	111 (24)
Fibrinogen (g/L)						
<2.5	565	37 (2.32)	528	264	14 (2.34)	250
2.5–<3	652	67 (3.89)	585	494	38 (3.58)	456
3–<3.5	512	62 (4.94)	450	397	52 (6.74)	343
3.5–<4	217	49 (10.12)	168	222	34 (8.36)	188
≥4	126	28 (11.20)	98	132	24 (10.75)	108
Missing	95			194		
Mean (SD)		3.18 (0.71)	2.87 (0.65)		3.29 (0.64)	3.05 (0.67)

*Unless otherwise stated. Rates are per 1000 person-years.

**Table 2:** Multivariable associations with cardiac mortality using baseline characteristics

	Age-adjusted		Fully adjusted*		
	HR	95% CI	HR	95% CI	p Value
Peak flow rate (L/min)†					
<430	1.54	1.07 to 2.21	1.53	1.04 to 2.25	0.025‡
430–<490	1.40	0.98 to 2.00	1.43	0.99 to 2.08	
490–<550	1.30	0.93 to 1.81	1.31	0.93 to 1.86	
≥550	1	–	1	–	
Systolic blood pressure					
Per 10 mm Hg increase	1.18	1.12 to 1.24	1.13	1.07 to 1.19	<0.0001
Body mass index					
Per kg/m^2^ increase	1.11	1.06 to 1.15	1.09	1.04 to 1.14	0.0002
Smoking status					
Non-smoker	1	–	1	–	0.015‡
Ex-smoker	1.24	0.84 to 1.85	1.04	0.70 to 1.56	
Current smoker	1.61	1.13 to 2.31	1.51	1.02 to 2.24	
Previous angina					
No	1	–	1	–	0.010
Yes	2.11	1.36 to 3.27	1.83	1.16 to 2.90	
Cholesterol					
Per mmol/L increase	1.29	1.17 to 1.43	1.18	1.05 to 1.33	0.005
Fibrinogen (g/L)					
≤3.5	1	–	1	–	0.001‡
>3.5–4	1.78	1.29 to 2.46	1.44	1.03 to 2.03	
>4	1.91	1.25 to 2.89	1.91	1.22 to 2.98	
Factor VII (% of standard)					
Per 10 unit increase	1.10	1.05 to 1.14	1.05	1.00 to 1.10	0.058
ECG					
Low risk	1	–	1	–	0.021
High risk	1.74	1.23 to 2.45	1.52	1.07 to 2.19	

*Also adjusted for age, triglycerides, platelets, white blood cell count, red blood cell count, haemoglobin, packed cell volume, alcohol use, social class, factor V and factor VIII.

†Unadjusted HRs for peak flow rate are 3.33 (<430), 2.51 (430–<490) and 1.70 (490–<550) compared with ≥550 L/min.

‡Trend test across groups.

In the secondary analysis using updated values for those in whom a follow-up visit was available, table 3 shows that the association of PFR with CHD mortality was less than for those with baseline values (trend test p=0.50), though the hazard was 15% higher in the lowest quartile of PFR than in the highest quartile (≥550 L/min). There was no evidence for an association of factor VII with CHD mortality, whereas the impact of a ‘high-risk’ ECG abnormality was stronger than when considering baseline ECG alone.

**Table 3:** Multivariable associations with cardiac mortality using time-updated model

	Age-adjusted		Fully adjusted*		
	HR	95% CI	HR	95% CI	p Value
Peak flow rate (L/min)^†^					
<430	1.36	0.94 to 1.97	1.15	0.77 to 1.70	0.50^‡^
430–<490	1.21	0.83 to 1.77	1.10	0.74 to 1.64	
490–<550	1.06	0.73 to 1.55	1.07	0.73 to 1.58	
≥550	1	–	1	–	
Systolic blood pressure					
Per 10 mm Hg increase	1.20	1.14 to 1.26	1.17	1.11 to 1.23	<0.0001
Body mass index					
Per kg/m^2^ increase	1.08	1.04 to 1.12	1.06	1.01 to 1.10	0.015
Smoking status					
Non-smoker	1	–	1	–	0.018^‡^
Ex-smoker	1.18	0.80 to 1.73	0.98	0.66 to 1.45	
Current smoker	1.74	1.21 to 2.51	1.50	0.99 to 2.25	
Previous angina					
No	1	–	1	–	0.001
Yes	2.05	1.41 to 2.97	1.93	1.31 to 2.83	
Cholesterol					
Per mmol/L increase	1.31	1.17 to 1.47	1.22	1.07 to 1.38	0.003
Fibrinogen (g/L)					
≤3.5	1	–	1	–	0.16^‡^
>3.5–4	1.37	0.98 to 1.92	1.04	0.73 to 1.48	
>4	1.82	1.26 to 2.63	1.37	0.92 to 2.04	
Factor VII (% of standard)					
Per 10 unit increase	1.07	1.03 to 1.13	1.03	0.98 to 1.09	0.22
ECG					
Low risk	1	–	1	–	<0.0003
High risk	2.04	1.55 to 2.68	1.71	1.28 to 2.28	

*Also adjusted for age, triglycerides, platelets, white blood cell count, red blood cell count, haemoglobin, packed cell volume, alcohol use, social class, factor V and factor VIII.

†Unadjusted HRs for peak flow rate are 3.18 (<430), 2.37 (430–<490) and 1.76 (490–<550) compared with ≥550 L/min.

‡Trend test across groups.

Further analyses considered complete-case analyses only, and also by imputation of the median values of missing variables. Results were largely consistent with those shown. Finally, an analysis was undertaken allowing for the potential competing risk of non-cardiac mortality and results were again consistent with the results presented (results not shown).

## Discussion

The prospective studies that have included measures of impaired lung function such as PFR, FEV or FEV_1_ and that have shown an association with the incidence of fatal CHD have come from many settings, often the USA,^4^ ^5^ ^7^ ^10^ but also from the UK and other European countries,^2^ ^6^ ^8^ ^12^ ^14^ China,^11^ ^15^ Australia and New Zealand,^9^ and India.^13^ Some have collected data from more than one country.^1^ ^3^ Many studies have typically reported relative risks (RRs) of 2 or less for the association between measures of impaired lung function and CHD, though in two North American studies,^7^ risks up to 4 were recorded. A systematic review of the relationship between FEV_1_ and cardiovascular mortality reported a pooled RR of 1.75 comparing those in the lowest quintile to the highest quintile of FEV_1_.^16^ In NPHS-1, the apparent effect of PFR on CHD, showing a HR difference of 53% between the lowest and highest quartiles, was modest using baseline characteristics (ie, analysis based on all participants) and had largely disappeared based on those who had follow-up examinations.

There do not appear to be large or consistent differences between FEV, FEV_1_ and PFR that would affect the findings of epidemiological studies. A study of older participants in east Boston^5^ also used only PFR, and gave much the same results as ours and as those for other studies (differences may be important in assessing lung function in clinical settings, such as acute asthma attacks in children.)

Data for men who had previously had major CHD events were not included in the analyses. However, abnormal ECGs often reflecting early pathological changes of CHD were, not surprisingly, significantly associated with CHD mortality. Omission of ECG data from other studies may thus well account for the generally stronger associations of PFR and other measures of impaired lung function with CHD than in NPHS–1, since these other studies have not been in a position to allow for the early pathology of CHD and the correlation of PFR with ECG findings. Smoking, cholesterol and systolic blood pressure were other independent risk factors associated with CHD death. We are aware of only one other study that has included measurement of plasma fibrinogen, which increases with the level of smoking but remains a strong independent risk factor for CHD. However, this study,^30^ CARDIA, dealt mainly with the association of fibrinogen with risk factors including COPD that contribute to CHD, rather than prospective associations with CHD itself.

In the present study, the association of plasma fibrinogen with fatal CHD was highly statistically significant in the multivariable analysis (table 2), which takes account of correlations between different risk factors. An explanation might be the contribution of fibrinogen to thrombosis in CHD, or as a marker of the degree of an inflammatory process in COPD and CHD. The trend was similar but reduced in the time-updated model (table 3), although there was little evidence for an association. There was a suggestion that factor VII may be associated with CHD in the multivariable model using baseline data, and may thus have contributed to the thrombotic element in CHD, though the finding was at a marginal level and there was no association in the time-updated model. There was no evidence that factors V and VIII and platelet count (as other components of the haemostatic system) were associated with fatal CHD. The Whitehall Cohort II study concluded that socioeconomic differences were not important in explaining the association between lung function and mortality.^12^

Virtually all prospective studies are missing data for some variables. Long-term follow-up periods almost inevitably mean that their data will be subject to competing risks of non-cardiac mortality. However, few allow for these points. We have used well-tested and accepted methods of imputation to allow for missing values in our data, and the results were similar compared with the analyses not using these methods. Allowance for competing risks of non-CHD mortality also made little difference.

Treatment of COPD appears to reduce the onset of cardiovascular disease.^31^ However, Lange *et al*^6^ concluded that “although impaired ventilatory function is a significant predictor of death from myocardial infarction and other cardiovascular diseases, it should not be regarded as a genuine risk factor for ischaemic heart disease.” We would agree with this view, in that the inclusion of a wide range of characteristics associated with CHD, including fibrinogen and abnormal ECG findings, suggests that measures such as PFR are not strongly and independently associated with fatal CHD, and that the view that they usefully predict CHD should be modified accordingly.

## References

[1] Friedman GD Klatsky AL Siegelaub AB Lung function and risk of myocardial infarction and sudden cardiac death. N Engl J Med 1976;294:1071–5 1256523 10.1056/NEJM197605132942001

[2] Menotti A Mariotti S Seccareccia S The 25 year estimated probability of death from some specific causes as a function of twelve risk factors in middle aged men. Eur J Epidemiol 1988;4:60–7 3356235 10.1007/BF00152694

[3] Ebi-Kryston KL Hawthorne VM Rose G Breathlessness, chronic bronchitis and reduced pulmonary function as predictors of cardiovascular disease mortality among men in England, Scotland and the United States. Int J Epidemiol 1989;18:84–8 2722386 10.1093/ije/18.1.84

[4] Marcus EB Curb JD MacLean CJ Pulmonary function as a predictor of coronary heart disease. Am J Epidemiol 1989;129:97–104 2910076 10.1093/oxfordjournals.aje.a115128

[5] Cook NR Evans DA Scherr PA Peak expiratory flow rate and 5-year mortality in an elderly population. Am J Epidemiol 1991;133:784–94 2021145 10.1093/oxfordjournals.aje.a115957

[6] Lange P Nyboe J Jensen G Ventilatory function impairment and risk of cardiovascular death and of fatal or non-fatal myocardial infarction. Eur Respir J 1991;4:1080–7 1756841

[7] Schroeder EB Welch VL Couper D Lung function and incident coronary heart disease: the Atherosclerosis Risk in Communities Study. Am J Epidemiol 2003;158:1171–81 14652302 10.1093/aje/kwg276

[8] Engstrom G Hedblad B Janzon L Reduced lung function predicts increased fatality in future cardiac events. A population-based study. J Intern Med 2006;260:560–7 17116007 10.1111/j.1365-2796.2006.01718.x

[9] Young RP Hopkins R Eaton TE Forced expiratory volume in one second: not just a lung function test but a marker of premature death from all causes. Eur Respir J 2007;30:616–22 17906084 10.1183/09031936.00021707

[10] Lee HM Le H Lee BT Forced vital capacity paired with Framingham Risk Score for prediction of all-cause mortality. Eur Respir J 2010;36:1002–6 20562119 10.1183/09031936.00042410

[11] Yang L Zhou M Smith M Body mass index and chronic obstructive pulmonary disease-related mortality: a nationally representative prospective study of 220,000 men in China. Int J Epidemiol 2010;39:1027–36 20400495 10.1093/ije/dyq051

[12] Sabia S Shipley M Elbaz A Why does lung function predict mortality? Results from the Whitehall II Cohort Study. Am J Epidemiol 2010;172:1415–23 20961971 10.1093/aje/kwq294 PMC2998200

[13] Hebert JR Pednekar MS Gupta PC Forced expiratory volume predicts all-cause and cancer mortality in Mumbai, India: results from a population-based cohort study. Int J Epidemiol 2010;39:1619–27 20846948 10.1093/ije/dyq157 PMC3031342

[14] Wannamethee SG Shaper AG Rumley A Lung function and risk of type 2 diabetes and fatal and nonfatal major coronary heart disease events: possible associations with inflammation. Diabetes Care 2010;33:1990–6 20519659 10.2337/dc10-0324 PMC2928349

[15] Smith M Zhou M Wang L Peak flow as a predictor of cause-specific mortality in China: results from a 15-year prospective study of ∼170,000 men. Int J Epidemiol 2013;42:803–15 23918851 10.1093/ije/dyt079

[16] Sin DD Wu L Man SF The relationship between reduced lung function and cardiovascular mortality: a population-based study and a systematic review of the literature. Chest 2005;127:1952–9 15947307 10.1378/chest.127.6.1952

[17] Wannamethee SG Shaper AG Ebrahim S Respiratory function and risk of stroke. Stroke 1995;26:2004–10 7482639 10.1161/01.str.26.11.2004

[18] Hozawa A Billings JL Shahar E Lung function and ischemic stroke incidence: the Atherosclerosis Risk in Communities study. Chest 2006;130:1642–9 17166977 10.1378/chest.130.6.1642

[19] Zureik M Kauffmann F Touboul PJ Association between peak expiratory flow and the development of carotid atherosclerotic plaques. Arch Intern Med 2001;161:1669–76 11434800 10.1001/archinte.161.13.1669

[20] Meade TW Brozovic M Chakrabarti RR Haemostatic function and ischaemic heart disease: principal results of the Northwick Park Heart Study. Lancet 1986;2:533–7 2875280 10.1016/s0140-6736(86)90111-x

[21] Brozovic M Stirling Y Harricks C Factor VII in an industrial population. Br J Haematol 1974;28:381–91 4441467 10.1111/j.1365-2141.1974.tb00819.x

[22] Brozovic M Chakrabarti R Stirling Y Factor V in an industrial population. Br J Haematol 1976;33:543–50 1009027 10.1111/j.1365-2141.1976.tb03573.x

[23] Meade TW Cooper JA Stirling Y Factor VIII, ABO blood group and the incidence of ischaemic heart disease. Br J Haematol 1994;88:601–7 7819072 10.1111/j.1365-2141.1994.tb05079.x

[24] Fearnley GR Measurement of spontaneous fibrinolytic activity. J Clin Pathol 1964;17:307–9 14159467 10.1136/jcp.17.3.307 PMC480754

[25] Rose GA Blackburn H Cardiovascular survey methods. East Afr Med J 1969;46:220–7 5345960

[26] Miall WE Greenberg G Mild hypertension—is there pressure to treat? An account of the MRC trial: Press Syndicate of the University of Cambridge, 1987

[27] Sperrin M Buchan I Modelling time to event with observations made at arbitrary times. Stat Med 2013;32:99–109 22807157 10.1002/sim.5509

[28] White IR Royston P Wood AM Multiple imputation using chained equations: issues and guidance for practice. Stat Med 2011;30:377–99 21225900 10.1002/sim.4067

[29] Fine JP Gray RJ A proportional hazards model for the subdistribution of a competing risk. J Am Stat Assoc 1999;94:496

[30] Thyagarajan B Jacobs DR Apostol GG Plasma fibrinogen and lung function: the CARDIA Study. Int J Epidemiol 2006;35:1001–8 16554379 10.1093/ije/dyl049

[31] Stone IS Barnes NC Petersen SE Chronic obstructive pulmonary disease: a modifiable risk factor for cardiovascular disease? Heart 2012;98:1055–62 22739636 10.1136/heartjnl-2012-301759

